# Network Anonymity and Cyberbullying among Chinese Adolescents: A Moderated Mediation Model

**DOI:** 10.3390/ijerph19020637

**Published:** 2022-01-06

**Authors:** Lingbo Zhao, Yingru Wu, Xiayu Huang, Lin Zhang

**Affiliations:** 1 Department of Applied Psychology, School of Humanities and Social Sciences, Fuzhou University, Fuzhou 350108, China; zlb@fzu.edu.cn (L.Z.); wuyingruchn@163.com (Y.W.); xiayu_huang@126.com (X.H.); 2 School of Psychology, Central China Normal University, Wuhan 430056, China; 3 Key Laboratory of Human Development and Mental Health of Hubei Province, Wuhan 430056, China; 4 Key Laboratory of Adolescent Cyberpsychology and Behavior, Ministry of Education, Wuhan 430056, China

**Keywords:** cyberbullying behavior, network anonymity, network morality, self-control, moderated mediation

## Abstract

Cyberbullying is an important issue which prevails among children and adolescents. The present study aimed to investigate the association between network anonymity and cyberbullying behavior and examine the mediating role of network morality and the moderating role of self-control in the linkage of network anonymity and cyberbullying behavior. A total of 620 participants were recruited from three high schools in southeast China and were required to complete a questionnaire measuring network anonymity, cyberbullying behavior, network morality, and self-control. A moderated mediation model was conducted by using PROCESS Macro for SPSS 3.5. The results showed that network anonymity was negatively associated with cyberbullying behavior among Chinese adolescents. Network morality mediated the association and self-control moderated the indirect association between network anonymity and cyberbullying behavior via network morality. These findings indicate that improving the network morality and self-control of adolescents with the joint efforts of individuals, families, government, and society as a whole may be an effective intervention strategy for cyberbullying behavior under the framework of digital citizens.

## 1. Introduction

Traditional bullying refers to a power imbalance between the bully and the victim, and the bully has a certain purpose and repeatedly attacks the victim [1]. Cyberbullying can be said to be an extension of traditional bullying through online platforms, which refers to individuals or groups communicating by sending electronic messages or other ways, in order to attack and harm vulnerable groups repeatedly who cannot protect themselves on the Internet [2]. In other words, cyberbullying is a form of repeated harassment through cybertechniques [3]. Cyberbullying prevails among children and adolescents, with some previous studies showing that approximately 75% of children and adolescents experience it [4]. The consequence of cyberbullying is often found to be worse than that of traditional bullying. A previous study found that Internet victims predict worse outcomes than traditional victims in terms of depression, anxiety, self-esteem problems, absenteeism, and physical health, and have a stronger association with suicidal ideation [5].

Teenagers are more susceptible to cyberbullying. For one thing, teenagers make up the majority of Internet users. According to the survey results of the 46th China Internet Network Information Center (CNNIC), as of June 2020, China’s Internet population reached 940 million. About 326 million of them were teenagers, accounting for 34.68% of the total, which was equal to one-third of Internet users. Another aspect is that adolescence is a critical period in the transition from a child to an adult, a time when adolescents are not only vulnerable to external influences but also immature in their self-control [4]. Previous studies have found that this age group is the primary one most likely to be bullied [6,7]. Therefore, it is crucial to investigate the influence factors and mechanism of cyberbullying behavior among teenagers, which may help provide society, schools, parents, and researchers with a better understanding of cyberbullying and adopt effective strategies to prevent negative consequences.

### 1.1. Theoretical Base

The theoretical base of the present study is the framework of digital citizenship proposed by Ribble [8]. According to the framework, individuals perceive greater anonymity in the online world because they have higher self-protection measures, such as encrypting their privacy, leaving less important information online, and feeling safer in cyberspace, which induces less cyberbullying behavior [9].

The concepts of “Respect” and “Protection” in digital citizenship also emphasize the need for individuals to use the Internet correctly and responsibly, to be kind to others, and to abide by the rules and laws of cyberspace, which indicates a lower risk of cyberbullying behavior [10].

### 1.2. Perceived Anonymity and Cyberbullying Behavior

The perceived anonymity of the web (also known as perceived anonymity) refers to the subjective anonymity that the actor perceives himself or herself to have when using the web [11]. There has been disagreement on the relationship between perceived anonymity and cyberbullying behavior. Many researchers considered anonymity as a situational risk factor of aggressive behavior and found that there was a positive relationship between anonymity and cyberbullying behavior [12,13,14].

However, other researchers claimed a possible negative association between perceived anonymity and cyberbullying behavior in China. Students in China have to attend some lessons on Internet literacy, which indicates that they may identify themselves with their role as digital citizens in the anonymous online world, which may be negatively correlated with the degree of cyberbullying behavior [15]. Moreover, China is a country that the network real-name system has been largely achieved, which could change the nature of the phenomenon. Anonymity is a kind of psychological perception of identity anonymity in China, which may increase the degree of safety and decrease the degree of impulsivity, and further decrease the degree of cyberbullying behavior [2,16].

### 1.3. Network Morality as a Mediator

Network morality may play a mediating role between perceived anonymity and cyberbullying behavior. First, perceived anonymity may be positively associated with network morality. The anonymity and invisibility of cyberspace make any behavior “physically isolated”, that is, the individual does not have to face others directly, and reduces the external pressure of moral evaluation, such as being considered to be meddlesome, to predict the individual’s network morality positively [17,18].

Meanwhile, network morality may be negatively associated with cyberbullying behavior. Cyberbullying perpetrators have a tendency to minimize their negative consequences to victims through moral disengagement [19]. Individuals who have a low degree of morality may participate in more aggressive behavior [4]. Many previous studies have empirically proven a strong association between moral disengagement and cyberbullying under certain conditions among youth [20,21,22].

### 1.4. Self-Control as a Moderator

Network morality may decrease the risk of cyberbullying behavior, as mentioned above. However, not all individuals with a high degree of network morality have a low risk of cyberbullying behavior, in which self-control may play an important role. Some researchers considered that individuals with a high degree of self-control would deliberate the consequence of cyberbullying behavior, which makes them rarely commit cyberbullying [16]. This deliberation is not necessarily based on moral considerations, e.g., individuals may be afraid of breaking the law or in the result of a defensive or retaliation response by another [16].

For individuals with a low degree of self-control, a negative association between network morality and cyberbullying behavior may be significant. According to the theory of idiosyncratic moral disengagement, when an individual’s self-control ability is weak and their level of network morality is lower, the individual more easily displays the network aggressive behavior [23,24].

### 1.5. Present Study

**Figure 1:**
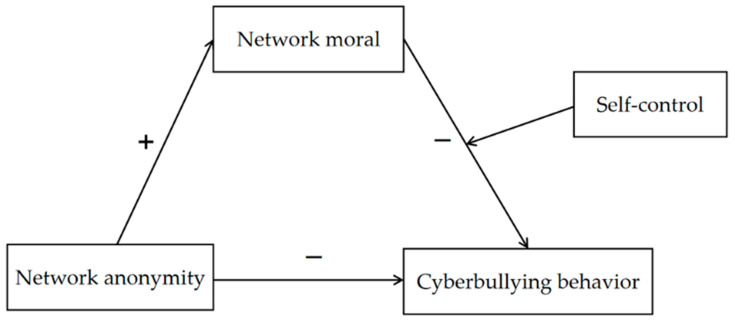
Hypothetical model.

Overall, considering the particularity of network anonymity in China, the present study aimed to investigate the direct effect of network anonymity on cyberbullying among adolescents in China first. In addition, the present study also aimed to examine the mediating role of network morality in the association between network anonymity and cyberbullying and the moderating role of self-control between network morality and cyberbullying. Specifically, our hypotheses are as follows (see Figure 1):

**Hypothesis** **1.** *Network anonymity would be negatively associated with cyberbullying behavior among Chinese adolescents.*

**Hypothesis** **2.** *Network morality would mediate the relationship between network anonymity and cyberbully behavior.*

**Hypothesis** **3.** *Self-control would moderate the effect of the network morality on cyberbullying behavior among adolescents, that is, for the individuals who have a low level of self-control, the lower the network morality, the more likely the cyberbullying behavior occurs.*

## 2. Materials and Methods

### 2.1. Participants

A total of 620 participants were recruited from three high schools in Southeast China by convenient sampling. Five hundred and twelve (82.7%) questionnaires were valid after screening according to the following exclusion criteria: (1) participants never used social networks or seldom used them, (2) participants did not fill in questionnaires completely. These 513 adolescents included 317 women (61.79%) and 196 men (38.21%), with an average age of 16.01 years old (age range: 14–18 years). Two hundred and sixty-six students (51.9%) had used the Internet for one to five years, 198 (38.6%) students had used the Internet for six to ten years, 38 (7.4%) students had used the Internet for 11–15 years, and 11 (2.1%) students had used the Internet for more than 16 years (see Table 1).

The present study was approved by the Ethics Committee of Department of Applied Psychology, School of Humanities and Social Sciences, Fuzhou University. Participants were free to quit the study at any time without consequence. All the data were only used for research and collected on a strictly voluntary and anonymous basis. All participants, schools, and their parents gave their consent to participate in the study.

### 2.2. Measures

Basic information regarding the age, gender, and years of Internet use of participants was collected. Besides the basic information, participants were also requested to fill out the questionnaires measuring the following variables. All variables were considered as manifest variables. The scale score for each scale was calculated by using the total score or average score.

#### 2.2.1. Outcome

Cyberbullying was regarded as the dependent variable in the present study and measured using the cyberbullying scale developed by Xu [25]. The cyberbullying scale is a 12-item, 5-point Likert scale (1 = *never*, 5 = *always*), which contains two subscales named direct cyberbullying and indirect cyberbullying. A high total score means a high level of cyberbullying behaviors. A sample item of direct cyberbullying was “I have made nicknames and made fun of others on the Internet”. A sample item of indirect cyberbullying was “I used others’ personal accounts and passwords without permission”. In the present study, the internal consistency for the scale was acceptable (Cronbach’s α = 0.76). The reliability of the scale in this study was 0.74.

#### 2.2.2. Independent Variable

Perceived anonymity was the independent variable and was measured using the perceived anonymity scale developed by Jung et al. [26]. It is a 4-item, 7-point Likert scale (1 = *strongly disagree*, 7 = *strongly agree*). A high total score indicates respondents perceived a high level of network anonymity. A sample item was “People cannot locate me in real life based on my information and messages in Cyworld”. In the present study, the internal consistency for the scale was acceptable (Cronbach’s α = 0.78). The reliability of the scale in this study was 0.77.

#### 2.2.3. Mediating Variable

Network morality was considered as the mediating variable in the present study and measured using the network morality scale [27]. It is a 9-item scale, which contains four subscales named network morality cognition (e.g., “Although the network society is virtual, it should also have its general moral”), network morality emotion (e.g., “If possible, I am willing to initiate a romantic relationship with an online friend”), network morality assessment (e.g., “Cyber hackers and their actions should be combated, not worshipped”), and network morality behavior (e.g., “Even if others do not know, I will not plagiarize on the Internet”). A high total score indicates a high level of network morality. In the present study, the internal consistency for the scale was acceptable (Cronbach’s α = 0.67). The reliability of the scale in this study was 0.66.

#### 2.2.4. Moderating Variable

Self-control was measured using the Chinese version of the self-control scale [28]. It is a 19-item, 5-point Likert scale (1 = *not at all like me*, 5 = *very much like me*), measuring the self-control of respondents from the following five perspectives: impulse control (e.g., “People would describe me as impulsive”), work or study performance (e.g., “I am able to work effectively toward long-term goals”), health habits (e.g., “I have a hard time breaking bad habits”), self-strained entertainment (e.g., “I do certain things that are bad for me, if they are fun”), and temptation resist (e.g., “I am good at resisting temptation”). In the present study, the internal consistency for the scale was good (Cronbach’s α = 0.85). The reliability of the scale in this study was 0.82.

### 2.3. Procedure

The self-administered online survey was conducted in southeast China from 26 September to 30 October 2020. Four classes were randomly selected out of each high school. Researchers informed participants of the purposes of the study, definitions of the main variables, that there were no right or wrong answers, and participants’ rights and obligations. All the students in the selected classes were required to fill out the questionnaire voluntarily under the supervision of the class teachers and research assistants in 15 min.

### 2.4. Data Analysis

IBM SPSS Statistics for Windows, Version 27.0 (IBM, Chicago, IL, USA), and PROCESS Macro for SPSS 3.5 (Andrew F. Hayes, Calgary, Canada) were utilized for data analyses. The specific procedure of data analyses was as follows: descriptive and correlation analyses were conducted for all variables, followed by examination of the common method bias using Harman’s single-factor test. Then, the mediating role of network morality and the moderating role of self-control in the relationship between network anonymity and cyberbullying were examined when controlling for gender by using the Model 14 of PROCESS. To test statistical significance, 95% confidence intervals of the bias-corrected boot-strapped method based on 5000 samples were used.

## 3. Results

### 3.1. Common Method Bias Test

Harman’s single-factor test showed that the variance of the first factor was 15.98%, less than the critical value (i.e., 40%), which indicated no serious common method bias in the present study.

### 3.2. Descriptive Statistics and Correlation Analyses

As shown in Table 2, self-control, network morality, and network anonymity were negatively correlated with cyberbullying behavior, i.e., Hypothesis 1 was verified. Network anonymity was positively correlated with network morality. There was no significant correlation between network anonymity and self-control. There was no significant correlation between network morality and self-control, either.

### 3.3. Test of Moderated Mediation

To test the hypothesized model, we used SPSS macro PROCESS, which is able to test the moderated mediation in a single model and has been used by numerous researchers [29]. First, we calculated the effect of perceived anonymity on cyberbullying. Then, we added network morality as a mediator. Last, we evaluated the moderating role of self-control. After controlling for gender, we examined the moderated mediating model by using Model 14 of PROCESS Macro for SPSS 3.5. The results showed that network anonymity was positively associated with network morality (β = 0.81, *p* < 0.01). In the moderated mediation model, there was no significant association between network anonymity and cyberbullying behavior (β = 0.01, *p* = 0.78). Network morality (β = −0.08, *p* < 0.01) and self-control (β = −0.1, *p* < 0.01) were negatively associated with cyberbullying behavior. The interaction between network morality and self-control was positively associated with cyberbullying behavior (β = 0.01, *p* < 0.01).

When the mediating variable was included, the direct effect of network anonymity on cyberbullying behavior was not significant. Network anonymity had a positive association with network morality (β = 0.81, *t* = 13.46, *p* < 0.01). Network morality had a significant negative association with cyberbullying (β = −0.08, *t* = −3.40, *p* < 0.01) In addition, the upper and lower bounds of the bootstrap 95% confidence interval of the mediating effect of network morality did not contain zero, indicating that network morality can play a mediating role in the linkage of network anonymity and cyberbullying behavior, i.e., hypothesis 2 was verified. The overall fitting index of the moderated mediation model was 0.23 (see Table 3).

**Figure 2:**
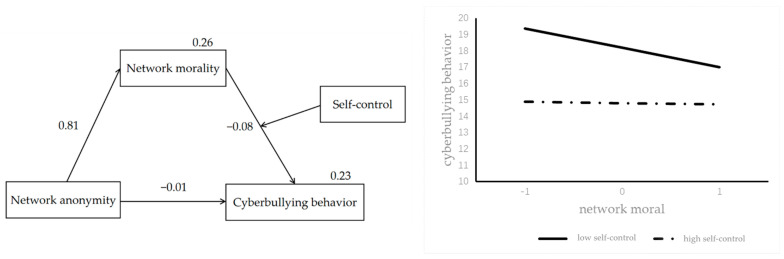
The model with indices (**left**) and the moderating role of self-control in the linkage of network morality and cyberbullying behavior (**right**).

Self-control score and Internet morality score were divided into high and low groups according to ±1 standard deviation in order to examine the moderated mediation effect. The results showed that self-control had a significant moderating effect. As shown in Table 4, self-control had a significant moderating effect on the second half of the mediation path (i.e., network anonymity—network morality—cyberbullying behavior). The regression coefficient of the interaction between online morality and self-control was significant (β = 0.01, *t* = 2.99, *p* < 0.01, LLCI = 0.001, ULCI = 0.008), i.e., hypothesis 3 was verified. Figure 2 shows how self-control moderates the effect of network morality on cyberbullying behavior. Among adolescents with low self-control ability, adolescents with a high cyber moral level are less likely to engage in cyberbullying, but there is no such effect among adolescents with high self-control ability.

## 4. Discussion

The present study examined the association between network anonymity and cyberbullying among adolescents, further exploring the underlying mediating roles of network morality and the moderating role of self-control in the linkage of network anonymity and cyberbullying. We found that anonymity had no direct predictive effect on cyberbullying behavior, but it had an indirect effect on adolescents’ cyberbullying behavior through cyber moral. Self-control played a moderating role in the mediation between cyber moral and cyberbullying behavior, that is, in individuals with high self-control, network morality has no significant mediating effect between network anonymity and cyberbullying behavior, while network morality has a significant mediating effect in the linkage of network anonymity and cyberbullying behavior among individuals with low self-control. The results showed that self-control may reduce the negative effect of network morality and weaken the mediating effect of network morality.

Consistent with the hypothesis of this study, the present study found that network anonymity had a significant negative effect on adolescents’ cyberbullying behavior, which is a novelty of the present study and could be supported by a few previous studies. For example, Lee and Sanchez found that when anonymity was high, students were less likely to engage in cyberbullying [30]. In other words, with the reduction in personal information exposure on the Internet, students will experience a reduction in the likelihood of cyberbullying behavior. However, some researchers found the opposite results, in which network anonymity was positively associated with cyberbullying behavior [12,13,14]. One possible reason for the inconsistent findings was the differences in the definition of network anonymity across countries. In China, network anonymity tends to be of a subjective feeling of security. In the context of China’s online real-name system, Chinese teenagers know intellectually that if they do something online that breaks the law, they will be found and punished quickly. Chinese adolescents who presuppose they will commit cyberbullying are much less likely to feel safe online and much less likely to expose personal information online.

The result of the mediating analysis showed that network anonymity could predict network morality positively, which is consistent with a previous study [31]. Individuals with a high sense of digital citizenship know how to use the Internet properly in order to gain a high sense of network security and can protect their own information well; thus, they have a high degree of network anonymity. Individuals with a high level of digital citizenship may also have a high level of network morality, and they may not engage in cyberbullying, even in anonymous settings.

Cyber morality has a significant negative effect on the cyberbullying behavior of adolescents, which is consistent with the previous study and the hypothesis of this study, that is, the higher the cyber moral of adolescents, the less likely they are to engage in cyberbullying [32,33]. A previous study showed that adolescents with low levels of network morality are more likely to engage in moral disengagement, which leads to further cyberbullying behavior [34]. According to the trait moral disengagement theory, individuals with lower cyber morality are prone to engage in cyberbullying, while individuals with higher cyber morality are able to recognize the negative consequences of cyberbullying and control their cognitive impulses, to reduce the negative emotional experience, so as to inhibit the emergence of bullying [20,21,22]. We also found that the effect of perceived anonymity on cyberbullying behavior was completely mediated by cyber morals. That is, network anonymity could not directly predict the cyberbullying behavior of adolescents, but it can indirectly influence cyberbullying behavior via network morality. This result indicated that in the network anonymous environment, the enhancement of the network morality level could reduce the occurrence of cyberbullying behavior.

Consistent with previous studies and the hypothesis of this study, we found that self-control ability had a significant negative effect on adolescents’ cyberbullying behavior. A previous study found that self-control was related to aggression and bad behavior [35]. Specifically, the lower the self-control, the more likely the individual was to act impulsively and to make decisions with less consideration of consequences. Generally, adolescents were more likely to take part in cyberbullying compared with adults. However, adolescents with high self-control had better self-restraint ability and could actively resist the harmful factors in the external environment, thus reducing cyberbullying behavior.

We also found that when the level of self-control was low, the level of cyber morality had a significant impact on cyberbullying behavior. At this time, individuals with a low level of cyber morality were more likely to engage in cyberbullying. However, individuals with a high level of self-control would not easily conduct cyberbullying behavior, no matter whether the moral level of the network was high or low. A possible explanation was that individuals with a high level of self-control can control their own behavior better and are not easily influenced by some negative information [16]. The influence of network morality on bullying behavior is not so important for them. According to the feedback loop model of self-control, individuals with low self-control are less able to resist temptation and regulate their behavior. However, their self-control system may be activated under the regulation of network morality mechanisms, which may reduce the likelihood of cyberbullying behavior. For individuals with a high level of self-control, with the improvement of the network morality level, it is more and more difficult for individuals to detect the inconsistency between unethical behavior and moral standards, and the self-control system is more and more activated [23,24].

## 5. Implications and Limitations

In spite of the above findings, several limitations should be noted when explaining the results of the present study. First, the cross-sectional design is a limitation. That is, we cannot ascertain the causal sequence of variables, which could be examined by longitudinal studies in the future. Second, the self-report questionnaire method was utilized in the present study, which brought a potential issue of subjective self-report and recall bias. Further research could collect data from adolescents’ caregivers, friends, and teachers to examine our results. Third, convenience sampling may limit the generalizability of the results. Further research could expand the size and diversity of the sample. Fourth, we should be cautious when interpreting the results derived from the low value of R^2^ in the present study. Last, future studies could examine the gender difference in the moderated mediation model in a more nuanced way.

Based on our findings, we try to provide some practical recommendations. This study found that network morality played a mediating role and self-control played a moderating role in the linkage of network anonymity and adolescents’ cyberbullying behavior, which provided an interesting implication for the prevention and intervention of adolescents’ cyberbullying behavior. For example, it is possible to improve the cyber morality and self-control of cyber-bullies in order to reduce the frequency of bullying. Specifically, first, from the perspective of adolescents, adolescents need to protect themselves by encrypting important information and reducing personal information exposure online. They should also strengthen eHealth literacy, maintain physical and mental health, participate in meaningful social activities actively, and communicate with classmates and friends frequently in order to obtain positive peer support.

At the school level, schools should strengthen the network morality education improve the network morality level of students. School leaders should ensure that they are setting an example of digital citizenship, that students in schools are safe, and that students learn the skills to live in the online world. By providing this knowledge, leaders should ensure that issues such as cyberbullying are reduced [8]. Schools should establish campus intervention groups and construct a good teacher–student atmosphere, where bullied students would feel comfortable reporting bullying behaviors to their tutors and school managers. Schools should fully encompass the role of psychological counseling centers to provide psychological assistance to students who are victims of traditional and cyberbullying to help them recover and build up their self-confidence. Schools can also improve mental health literacy and build websites to introduce the concept of cyberbullying, the harm of cyberbullying behavior, the prevention and treatment of cyberbullying, and other related knowledge.

In a parenting sense, parents can learn more knowledge about digital citizenship (e.g., digital literacy, digital etiquette, digital security, digital rights, and responsibilities of the cyber world). Parents should strengthen communication with young people and establish good parent–child relationships. Moreover, parents should actively respond to and cooperate with the digital citizenship education carried out by schools.

At the national and governmental level, first of all, countries should establish and improve the laws and regulations related the cyberbullying and advance the process of school bullying (including cyberbullying) legislation. Secondly, the government may think over the real-name system and provide a good strategy to ensure not only a clean and green Internet environment but also freedom of expression. Thirdly, the country should vigorously promote digital citizenship education. Lastly, the role of social media organizations should not be ignored, because it plays a role in advocacy guidance.

## 6. Conclusions

Overall, we can now state that network anonymity is negatively associated with cyberbullying among Chinese adolescents under the framework of digital citizenship. Moreover, the network morality mediated and the self-control moderated the association between network anonymity and cyberbullying. These findings may contribute to researchers and practitioners gaining a better understanding of cyberbullying in adolescents. Future interventions on cyberbullying can start with self-control and network morality. Under the framework of digital citizenship, the relationship between network anonymity and cyberbullying has been explained, which may give researchers a better understanding of the role of network anonymity in cyberbullying behavior.

## Figures and Tables

**Table 1:** Descriptive statistics of the participants.

Participant Attributes	N (%)/Mean (SD)
Gender	
Male	196 (38.21%)
Female	317 (61.79%)
Age	16.01 (1.83)
Internet age	
1–5 years	266 (51.9%)
6–10 years	198 (38.6%)
11–15 years	38 (7.4%)
More than 16 years	11 (2.1%)

**Table 2:** Correlation analysis of all continuous variables.

Variable	Mean	SD	1	2	3
1. Self-control	63.93	11.79	1		
2. Network morality	4.88	8.99	0.05	1	
3. Perceived anonymity	2.08	5.78	0.01	0.51 **	1
4. Cyberbullying behavior	16.52	4.41	−0.39 **	−0.17 **	−0.09 *

Note. N = 513. * p < 0.05, ** *p* < 0.01.

**Table 3:** Moderated mediation model.

Regression		Overall Fitting Index			Significance	
Outcome	Predictor	R	R^2^	F	β	t
Network morality		0.51	0.26	91.37		
	Network anonymity				0.81	13.46 **
	Gender				−0.80	−1.13
Cyberbullying behavior		0.48	0.23	30.41		
	Network anonymity				0.01	0.28
	Network morality (A)				−0.08	−3.40 **
	Self-control (B)				−0.15	−10.17 **
	A × B				0.01	2.99 **
	Gender				−1.87	−5.19 **

Note. N = 513. ** *p* < 0.001.

**Table 4:** Total, individual, and serial indirect effects for self-control on cyberbullying behavior and bias-corrected 95% confidence intervals.

Level of Self-Control	Effect	BootSE	BootLLCI	BootULCI
Low	−0.10	0.04	−0.19	−0.03
High	−0.01	0.03	−0.05	0.04

## Data Availability

The data presented in this study are available on request to the authors. Some variables are restricted to preserve the anonymity of study participants.
